# Editorial: Maintain that brain - protecting and boosting cognitive flexibility

**DOI:** 10.3389/fnhum.2023.1279374

**Published:** 2023-09-12

**Authors:** Barbara Colombo, Anna-Katharine Brem, Joukje Oosterman

**Affiliations:** ^1^Behavioral Neuroscience Lab, Champlain College, Burlington, VT, United States; ^2^Department of Old Age Psychiatry, Institute of Psychiatry, Psychology and Neuroscience, King's College London, London, United Kingdom; ^3^University Hospital of Old Age Psychiatry, University of Bern, Bern, Switzerland; ^4^Donders Institute for Brain, Cognition, and Behaviour, Radboud University, Nijmegen, Netherlands

**Keywords:** aging, creativity, brain health, cognitive flexibility, cognitive reserve

Studies have consistently demonstrated that cognitively stimulating activities can protect against the negative effects of brain pathology on cognition in aging (Brem and Sensi, 2018). These stimulating activities are thought to increase adaptive behavior via compensatory processes, a phenomenon also known as cognitive reserve (CR) (Barulli and Stern, 2013). CR is a fascinating concept because it has been proven to be linked to many different mechanisms: starting from general cognitive functions (Roldán-Tapia et al., 2012) and narrowing it down to creativity (Colombo et al., 2018) and more specifically to flexibility of thought (Fusi et al., 2021a). Cognitive flexibility serves as a foundational cognitive trait that enables individuals to adapt more effectively to a rapidly changing world. It empowers individuals to embrace uncertainty, learn from experiences, and navigate complex challenges. Enhancing cognitive flexibility can lead to heightened adaptability, ultimately improving an individual's ability to thrive in diverse environments and situations.

This path can be explained by reflecting on the fact that although it is generally accepted that more efficient executive functioning is a main mediator of this adaptive behavior, little is known regarding the exact underlying cognitive and neurophysiological mechanisms. One potential explanation is that increased adaptability can be explained via an underlying enhanced cognitive flexibility (Fusi et al., 2021a).

Papers included in this Research Topic embrace all the spectrum of components that allow to protect our brain, with a target focus on flexibility of thought, or cognitive flexibility.

If, as mentioned above, the relationship between CR and creativity has been reported in the literature, an understudied aspect is linked to some of the neurological bases of this association. An interesting insight into this is provided by the paper by Nobukawa et al. that focused on functional connectivity in older adults. Their results highlighted how older participants with high creativity exhibited high functional connectivity overall, with no significant effect of their IQ. This finding seems to support the idea that creativity entails widespread brain connectivity. Interestingly, but not surprisingly, a similar conclusion has been reported when focusing on the CR (Stampanoni Bassi et al., 2019). The paper by Khachatryan et al. using an oddball paradigm, follows and expands this line of research, by relating the P300 amplitude and latency to different proxies of cognitive reserve. Specifically, they found that education and leisure activities were linearly related to the P300 latency, whereas more complex, non-linear relations between the P300 amplitude and education and working activities were found. This study therefore identifies distinct neural components underlying different proxies that are presumed to increase reserve.

Because of the connection between CR and cognitive flexibility, and because of the fact that they tend to be widespread in the brain, strategies that aim to improve adaptability via the improvement of cognitive flexibility have been reported to translate best into preservation and enhancement of cognitive abilities and therefore successful aging (e.g., Buitenweg et al., 2017; Colombo et al., 2022).

Yet, the effect on some specific CR proxies have been investigated to a lesser extent. Some of these proxies include emotion regulation (Huang et al., 2019; Colombo et al., 2023) as well as behavior within different social situations (Ihle et al., 2018). The paper by Allaert et al. addresses this aspect in an interesting way, by using non-invasive brain stimulation (specifically, tDCS) as a form of intervention. Their results suggest that attenuated self-referential attention in specific emotion-inducing social situations may be a neurocognitive mechanism through which tDCS reduces emotional reactivity.

Another open question is how differently our brains cope with damage when pathology is present if compared to normal aging. A first answer can be found in the study by Jansen et al. that focuses on the effect of cognitive reserve on cognition in older adults with different levels of cognitive decline: from subjective cognitive complaints to Alzheimer's dementia. Their results demonstrate how with more advanced levels of cognitive impairment, the positive effects of cognitive reserve proxies on cognition become less pronounced. This suggests that our ability to adapt, presumably mediated via cognitive reserve, may be functioning best in case of relatively intact cognition or only mild levels of cognitive impairment. That being said, this relationship has been proven not to be linear, but to be most likely moderated by many other factors, like, for example, the time given to perform divergent thinking tasks (Fusi et al., 2021b). If the type of intervention and the way it is administered plays a role in the results, as indirectly suggested by the findings of Jansen et al. seeing how different interventions might affect brain coping mechanisms would be important.

The study by Stalpaert et al. provides a first answer with a specific reference to patients with Primary Progressive Aphasia. Their results highlight how patients who received speech-language therapy showed specific neurophysiological changes, suggesting that this therapy might be more effective for patients with a non-fluent variant of aphasia.

In summary, the Research Topic of studies published in this Frontiers Research Topic shed light on a range of understudied aspects of CR including the relationship between CR and other cognitive, behavioral, and neural components, the modulation of CR proxies through non-invasive brain stimulation, and the role of CR in therapeutic outcomes and in protective effects during pathological aging. While the relationship between CR and cognition is complex and moderated by various factors, promising results warrant further investigation of interventions targeting CR to enhance cognitive abilities.

## Author contributions

BC: Writing—original draft, Writing—review and editing. A-KB: Writing—original draft, Writing—review and editing. JO: Writing—original draft, Writing—review and editing.
